# Buffering the healthcare gap: the role of Korean medicine in unmet healthcare needs among care recipients

**DOI:** 10.3389/fmed.2026.1890354

**Published:** 2026-08-25

**Authors:** Minjung Park, Dongsu Kim, Lin Ang, Jennifer Hunter

**Affiliations:** 1 Department of Preventive Medicine, College of Korean Medicine, Gachon University, Seongnam, Republic of Korea; 2 Department of Preventive Medicine, College of Korean Medicine, Dongshin University, Naju, Republic of Korea; 3 Health Research Group, Sydney, NSW, Australia; 4 School of Pharmacy, Faculty of Medicine and Health, The University of Sydney, Sydney, NSW, Australia

**Keywords:** caregiving, fixed-effects panel model, Korea health panel, integrative healthcare, Korean medicine, unmet healthcare needs

## Abstract

**Background:**

Population aging is increasing reliance on formal and informal care. Studies consistently report care recipients experiencing higher levels of unmet healthcare needs, highlighting persistent barriers. The compensatory role of traditional medicine in addressing these barriers remains underexplored. This study examines the longitudinal association between care receipt and unmet healthcare needs and evaluates whether traditional Korean Medicine (KM) utilization moderates this association.

**Methods:**

We analyzed five waves (2014–2018) of the Korea Health Panel Survey (KHPS), yielding 66,188 person-year observations from individuals aged 19 and older. A conditional fixed-effects logit model with year fixed effects was employed to control for time-invariant unobserved heterogeneity and common temporal impacts. Unmet healthcare needs and key independent variables (receiving formal or informal care, KM utilization, and their interaction) were binary. Covariates were selected based on the Andersen Behavioral Model of Health Services Use. Estimation relied on within-person variation in unmet healthcare needs (5,165 individuals; 23,956 person-years), and results are reported as odds ratios (ORs) with 95% confidence intervals (CIs).

**Results:**

Receiving care was significantly associated with higher odds of unmet healthcare needs (OR = 1.28, 95% CI: 1.08–1.52, p = 0.005). KM utilization was not independently associated with unmet healthcare needs (OR = 1.02, 95% CI: 0.93–1.12). The interaction between receiving care and KM utilization was directionally consistent with a buffering association but did not reach statistical significance (OR = 0.90, 95% CI: 0.67–1.20).

**Conclusions:**

This study provides longitudinal evidence that adults who receive formal or informal care are more likely to report unmet healthcare needs, even within a system with universal healthcare coverage. Among care recipients, KM utilization was associated with a lower point estimate of unmet healthcare needs, in the direction of a buffering role; however, this interaction did not reach statistical significance and should be interpreted as suggestive. These findings highlight the healthcare access barriers faced by care recipients and suggest that the potential role of KM in meeting their complex healthcare needs warrants confirmation in larger samples with more detailed measures of KM utilization. Policymakers may consider strengthening the integration of KM into community-based care models as further evidence accumulates.

## 1 Introduction

Population aging is one of the most pressing demographic challenges worldwide, and South Korea is at the forefront of this transition (1). The proportion of the population aged 65 and older increased sharply from 7.2% in 2000 to 15.7% in 2020, and the demand for long-term care (LTC) services has grown correspondingly (2). Approximately 25.3% of older adults experience difficulties in activities of daily living required for self-care or instrumental activities of daily living required for independent community living, and this prevalence rises substantially among those aged 85 and older (3). The increasing number of older adults requiring LTC has prompted substantial policy responses, including the introduction of the public LTC-Insurance system in 2008, which provides both home-based and institutional care services to eligible beneficiaries, of whom the majority are older than 65 years (4).

In recent years, South Korea has expanded its LTC infrastructure through multiple policy initiatives (5). The Community Care program, launched in 2019, aims to integrate health and social welfare services at the local level (6). Additionally, home-visit medical care centers have been established to provide medical services directly to care recipients in their homes, and visiting care programs have been implemented to reach individuals who face barriers to accessing conventional healthcare facilities (7). Despite these efforts, the healthcare needs of older adults and individuals receiving LTC remain incompletely met, highlighting a persistent gap between LTC provision, insurance and healthcare access (8).

Unmet healthcare needs refer to situations in which individuals perceive a need for medical treatment or examination but are unable to receive it (9). These unmet needs represent a critical indicator of healthcare system performance (9). In South Korea, approximately 11.6% of the population experiences subjective unmet healthcare needs (10, 11). Previous research has identified multiple determinants of unmet healthcare needs, including socioeconomic status, health insurance type, chronic disease burden, functional limitations and geographically underserved communities (7, 11). Older adults requiring care, who often have complex health conditions and functional limitations, are particularly vulnerable to experiencing unmet healthcare needs. Ju et al. (7) found that older adults receiving LTC had significantly higher odds of unmet healthcare needs compared to those with no functional limitations and associated care needs; with an odds ratio of 2.76 for those accessing privately funded care and 5.84 for those accessing publicly funded care through the LTC-Insurance scheme.

For individuals requiring LTC, Korean Medicine (KM) may offer several advantages. Its emphasis on chronic disease management, pain relief, and functional rehabilitation may complement conventional medical services and help address healthcare gaps (12). KM, a traditional medical system encompassing acupuncture, herbal medicine, moxibustion, Chuna manual therapy, Pharmaco-acupuncture and other modalities, has long been an integral component of healthcare in South Korea (13). KM adopts a holistic approach that emphasizes the balance of bodily systems and the maintenance of health through preventive care (14). In South Korea, an estimated 25% of adults use KM each year, with higher use among older adults, women, and individuals with musculoskeletal disorders compared with those using conventional Western medicine (15). Reasons for using KM include perceived effectiveness, safety and quality of care, lower costs, closer proximity of services, and the social influence of reputation and family- or community-based recommendations (16). KM services are covered under the National Health Insurance (NHI) system and are widely accessible through a network of KM clinics and hospitals (17).

Despite the growing body of literature on LTC services and unmet healthcare needs, as well as on the effects of LTC-Insurance on healthcare utilization and outcomes (2, 4, 18, 19), the potential moderating role of KM utilization in the relationship between receiving care and unmet healthcare needs has not been investigated. Previous cross-sectional studies examining the association between KM use and unmet healthcare needs have been limited by their inability to control for unobserved individual heterogeneity, thereby leaving the causal direction ambiguous. To address these limitations, this study employs an individual fixed-effects panel regression model using five waves (2014–2018) of the longitudinal Korea Health Panel Survey (KHPS), a nationally representative longitudinal survey. By examining within-individual changes over time, the fixed-effects approach substantially controls for time-invariant confounders, such as health literacy, healthcare preferences, and personality traits, thereby providing more robust evidence regarding the moderating association of KM utilization on unmet healthcare needs among individuals living at home who are receiving formal or informal care.

## 2 Methods

### 2.1 Data source

This study utilized data from the Korea Health Panel Survey (KHPS), which is jointly administered by the Korea Institute for Health and Social Affairs (KIHASA) and the National Health Insurance Service (NHIS) (20). The KHPS is a nationally representative longitudinal survey that follows households and individuals randomly selected from the 2005 Korean Population and Housing Census using regional stratification variables. The panel has been conducted annually since 2008 and collects comprehensive information on healthcare utilization, health status, health behaviors, and socioeconomic characteristics (11).

**Figure 1:**
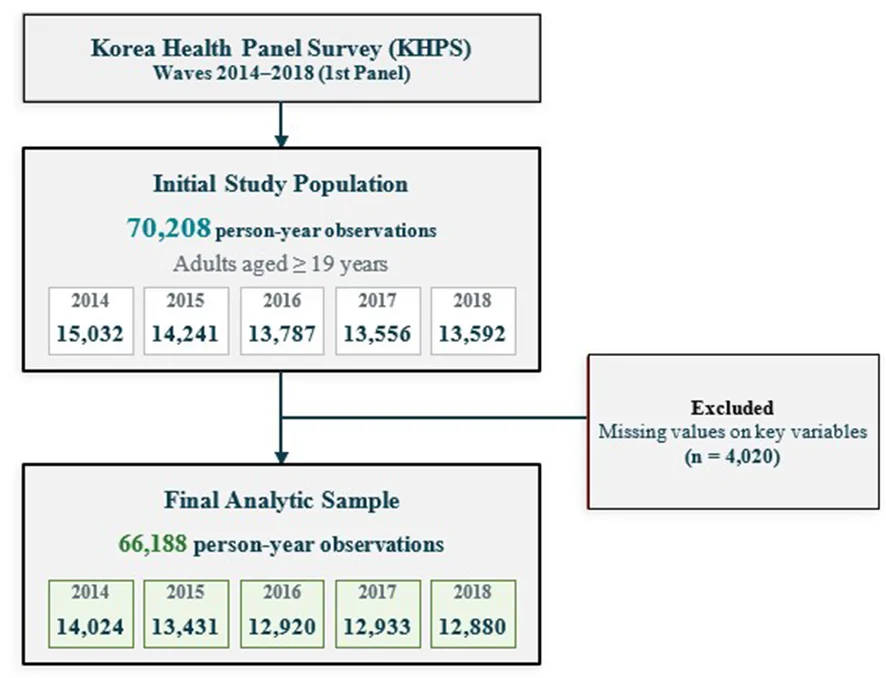
Flowchart of study population selection.

For this study, we used five waves of data from 2014 to 2018. The initial study population consisted of 70,208 person-year observations from individuals aged 19 years and older. The initial study population included 70,208 person-year observations (15,032 in 2014, 14,241 in 2015, 13,787 in 2016, 13,556 in 2017, and 13,592 in 2018). After excluding observations with missing values on key variables, the final analytic sample included 66,188 person-year observations. The distribution of observations by survey year was as follows: 14,024 in 2014, 13,431 in 2015, 12,920 in 2016, 12,933 in 2017, and 12,880 in 2018 (Figure 1).

### 2.2 Variables

#### 2.2.1 Dependent variable

In the KHPS questionnaire, a specific question was asked: “Have you ever experienced not receiving the necessary medical treatment or examination (excluding dental care) in the past year (12 months)?” Respondents had three answer options: Option (1): “Yes, I have experienced it at least once.” Option (2): “No, I have not experienced it” and Option (3): “No medical treatment or examination of any kind was needed.” Based on the responses, the individuals were categorized into two groups. Those who chose Option (1) were classified as the “Unmet Needs” group, while those who selected Option (2) or Option (3) were placed in the “non-Unmet Needs” group. Subsequently, a binary dependent variable was established, with a value of “1” for individuals in the “Unmet Needs” group and “0” for those in the “non-Unmet Needs” group. Therefore, having unmet healthcare needs means whether respondents reported needing medical treatment or examination during the survey year but being unable to receive it at least once. This definition is consistent with previous studies using the KHPS data (7, 11).

#### 2.2.2 Key independent variables

Care recipient was operationalized as a binary variable indicating whether the respondent received any formal or informal care services during the survey year. Formal care refers to professional services that may be funded through LTC-Insurance or private funding. Informal care refers to support provided by paid or unpaid caregivers, including family members, friends and neighbors. KM utilization was measured as a binary variable indicating whether individuals used any KM services during the survey year, including inpatient and outpatient visits to KM clinics or hospitals.

#### 2.2.3 Interaction term

The primary variable of interest was the interaction between care recipient status and KM utilization (Care recipient × KM utilization), which was included to assess whether KM utilization moderates the relationship between care recipient status and unmet healthcare needs.

#### 2.2.4 Covariates

Following the Andersen Behavioral Model of Health Services Use, covariates were categorized into three groups. Predisposing factors included age, sex, marital status (unmarried vs. married), employment status (non-manual, manual, and non-economic), region of residence (countryside, metropolitan, and capital), and educational attainment (middle school or below, high school, and university or above). Enabling factors included Health Insurance type (National Health Insurance, Medical Aid (for low-income citizens), and other) and household income quintile. Need factors included the number of chronic diseases, disability status (yes/no), and activity limitation (yes/no). This covariate framework is consistent with established approaches in the literature on healthcare utilization and unmet healthcare needs (3, 11).

### 2.3 Statistical analysis

To investigate the moderating association of KM utilization in the relationship between care receiving and unmet healthcare needs, we employed a conditional fixed-effects (FE) logit model with year fixed effects. The FE model controls for all time-invariant unobserved individual heterogeneity, thereby substantially mitigating potential omitted variable bias that could confound the estimated relationships [Cho and Kwon, (4); Kim and Mitra, (2)]. In the conditional likelihood, the within-person sum of the outcome serves as a sufficient statistic for the individual effect, which is eliminated rather than estimated; the estimator is therefore free from the incidental parameters bias that affects unconditional (dummy-variable) FE logit estimators in short panels. The econometric specification was:


$$\begin{array}{r} logit\left(Pr\left(UNMET_{it}=1\right)\right)=\beta_{1}CARE_{it}+\beta_{2}KM_{it}\\ +\beta_{3}{\left(CARE\times KM\right)}_{it}+\delta X_{it}+\gamma_{t}+\alpha_{i} \end{array}$$

where UNMET_*it*_ represents unmet healthcare needs for individual *i* at year *t*; CARE_*it*_ denotes receiving care status; KM_*it*_ indicates KM utilization; β_3_ captures the moderating association; X_*it*_ is a vector of time-varying covariates; γ_*t*_ denotes year fixed effects controlling for common temporal shocks (e.g., policy changes and macroeconomic conditions); and α_*i*_ represents individual-specific effects, which are conditioned out of the likelihood rather than estimated. No additive error term is specified because the model is estimated by conditional maximum likelihood. Coefficients were exponentiated and are reported as odds ratios (ORs) with 95% confidence intervals (CIs).

Western medicine (WM) utilization was excluded from the model specification despite its theoretical relevance. Given that the vast majority of the study population utilized WM at least once annually, WM utilization was effectively saturated at the population level, resulting in near-zero within-individual variation across waves. Because the FE estimator identifies coefficients exclusively from within-individual changes over time, including a variable with negligible within-person variation would substantially attenuate statistical power and render reliable coefficient estimation infeasible (21).

Consistent with the properties of individual FE models, time-invariant characteristics (e.g., sex) and variables perfectly collinear with the year effects (e.g., age) were automatically absorbed and omitted from estimation. Identification relies on within-person variation in the outcome: individuals whose unmet-need status did not change during the observation period contribute no information to the conditional likelihood. Of the 66,188 person-year observations, 5,165 individuals (23,956 person-years) exhibited within-person variation in unmet healthcare needs and identify the coefficients. The main model was estimated without survey weights, because the fixed-effects design absorbs time-invariant determinants of sample selection and the estimand is a within-person partial association rather than a population total. As sensitivity analyses, we re-estimated the model as an unconditional FE logit, both unweighted and applying the KHPS longitudinal weights normalized to a mean of one, with standard errors clustered at the individual level, and obtained virtually identical point estimates for the key variables (Supplementary Table S1). To assess potential multicollinearity among the independent variables, particularly between activity limitation and receiving formal or informal care, the Variance Inflation Factor (VIF) was calculated using the baseline data.

For the descriptive analysis, differences in baseline characteristics across survey years were evaluated using the Rao-Scott adjusted chi-square test for categorical variables and the design-based Kruskal–Wallis test for continuous variables. These tests account for the complex survey design and longitudinal weights of the KHPS. The KHPS provides longitudinal weights that are calibrated to adjust for sample attrition and non-response over successive waves, ensuring that the analytic sample remains representative of the national population across the study period. These weights incorporate the original sampling design, including stratification and clustering, and are updated at each wave to reflect changes in the panel composition.

All analyses were conducted using R software (version 4.5.2; R Foundation for Statistical Computing, Vienna, Austria). The conditional FE logit model was estimated using the survival package (clogit), and sensitivity analyses were estimated using the fixest package.

## 3 Results

### 3.1 Descriptive statistics

Table 1 presents the descriptive statistics for the study population across the five survey years (2014–2018). The proportion of individuals utilizing KM services remained relatively stable at 17% during 2014–2016 and increased modestly to 19% by 2017–2018 (*p* < 0.001). Approximately 3% of the sample reported receiving formal or informal care services each year, with no statistically significant change over time (*p* = 0.13).

**Table 1:** Descriptive statistics of study population (*N* = 66,188 person-years observations).

Variable/Year	2014	2015	2016	2017	2018	*p*-value
**KM utilization**						< 0.001
No	11,412 (83%)	10,925 (83%)	10,487 (83%)	10,292 (81%)	10,117 (81%)	
Yes	2,612 (17%)	2,506 (17%)	2,433 (17%)	2,641 (19%)	2,763 (19%)	
**Care recipient**						0.13
No	13,427 (97%)	12,795 (97%)	12,313 (97%)	12,249 (97%)	12,238 (97%)	
Yes	597 (2.8%)	636 (2.8%)	607 (3.0%)	684 (3.2%)	642 (2.9%)	
**Unmet healthcare needs**						< 0.001
No	12,208 (88%)	11,556 (87%)	11,471 (90%)	11,551 (89%)	11,303 (88%)	
Yes	1,816 (12%)	1,875 (13%)	1,449 (10%)	1,382 (11%)	1,577 (12%)	
**Age**, mean (SD)	58.48 (16.38)	57.59 (16.25)	57.29 (16.29)	56.67 (16.42)	56.10 (16.58)	< 0.001
**Sex**						0.5
Male	6,387 (47%)	6,116 (48%)	5,825 (47%)	5,835 (47%)	5,830 (48%)	
Female	7,637 (53%)	7,315 (52%)	7,095 (53%)	7,098 (53%)	7,050 (52%)	
**Marital status**						< 0.001
Unmarried	4,147 (33%)	4,104 (35%)	4,028 (35%)	4,170 (36%)	4,270 (37%)	
Married	9,877 (67%)	9,327 (65%)	8,892 (65%)	8,763 (64%)	8,610 (63%)	
**Employment**						< 0.001
Non-manual	2,640 (23%)	2,521 (24%)	2,490 (25%)	2,536 (25%)	2,567 (26%)	
Manual	5,846 (41%)	5,291 (39%)	5,201 (40%)	5,242 (41%)	5,301 (41%)	
Non-economic	5,538 (36%)	5,619 (37%)	5,229 (35%)	5,155 (34%)	5,012 (33%)	
**Region**						0.064
Rural	7,989 (54%)	7,652 (54%)	7,331 (53%)	7,418 (54%)	7,424 (54%)	
Metropolitan	4,194 (26%)	4,029 (26%)	3,926 (26%)	3,890 (26%)	3,868 (26%)	
Capital	1,841 (21%)	1,750 (21%)	1,663 (21%)	1,625 (20%)	1,588 (20%)	
**Education**						< 0.001
≤ Middle school	2,933 (14%)	2,775 (13%)	2,661 (13%)	2,611 (12%)	2,537 (12%)	
High school	6,006 (42%)	5,712 (41%)	5,442 (41%)	5,383 (41%)	5,299 (40%)	
≥ University	5,085 (45%)	4,944 (46%)	4,817 (46%)	4,939 (47%)	5,044 (48%)	
**Health insurance**						< 0.001
NHI	13,413 (97%)	12,838 (96%)	12,293 (96%)	12,295 (96%)	12,258 (96%)	
Medical Aid	435 (2.6%)	430 (2.7%)	454 (2.9%)	424 (2.7%)	415 (2.7%)	
Other	176 (0.8%)	163 (0.9%)	173 (0.9%)	214 (1.2%)	207 (1.3%)	
**Income quintile**						< 0.001
1st (lowest)	2,098 (11%)	1,992 (9.9%)	1,855 (9.4%)	1,827 (9.5%)	1,797 (9.1%)	
2^nd^	2,625 (17%)	2,482 (16%)	2,389 (16%)	2,348 (15%)	2,345 (15%)	
3^rd^	2,954 (21%)	2,837 (22%)	2,737 (22%)	2,738 (21%)	2,685 (21%)	
4^th^	3,123 (24%)	3,011 (25%)	2,910 (25%)	2,969 (26%)	2,992 (26%)	
5th (highest)	3,224 (28%)	3,109 (28%)	3,029 (28%)	3,051 (29%)	3,061 (29%)	
**No. of chronic diseases**, mean (SD)	1.50 (2.01)	1.50 (2.00)	1.57 (2.06)	1.47 (1.96)	1.56 (2.05)	< 0.001
**Disability**						0.2
No	13,061 (95%)	12,517 (95%)	12,014 (95%)	12,014 (95%)	11,960 (95%)	
Yes	963 (5.3%)	914 (5.1%)	906 (5.3%)	919 (5.4%)	920 (5.4%)	
**Activity limitation**						< 0.001
No	13,256 (96%)	12,440 (95%)	12,101 (95%)	12,229 (96%)	12,131 (96%)	
Yes	768 (4.1%)	991 (5.1%)	819 (4.7%)	704 (4.0%)	749 (4.2%)	

KM, Korean medicine; NHI, national health insurance. Values are *n* (weighted %) for categorical variables and mean (SD) for continuous variables. *p*-values based on Rao-Scott adjusted Chi-square test (categorical) and design-based Kruskal–Wallis's test (continuous).

The mean age of respondents decreased slightly from 58.48 years (SD = 16.38) in 2014 to 56.10 years (SD = 16.58) in 2018. The sex distribution was consistent across years, with females comprising approximately 52–53% of the sample. The proportion of married individuals declined from 67% to 63%, while the share of those with university-level education increased from 45% to 48%.

Regarding health-related characteristics, the mean number of chronic diseases fluctuated between 1.47 and 1.57 across survey years. The prevalence of disability was relatively stable, ranging from 5.1% to 5.4%, while activity limitation fluctuated between 4.0% and 5.1%.

Differences in most baseline sociodemographic and health-related characteristics across survey years were statistically significant, as evaluated using the Rao-Scott adjusted chi-square test for categorical variables and the design-based Kruskal–Wallis test for continuous variables.

### 3.2 Fixed-effects panel regression

Table 2 presents the results of the conditional fixed-effects logit model with year fixed effects. Of the 66,188 person-year observations in the analytic sample, 5,165 individuals (23,956 person-years) exhibited within-person variation in unmet healthcare needs and contributed to the conditional likelihood. All VIF values were well below the conventional threshold of 5, indicating no evidence of severe multicollinearity.

**Table 2:** Conditional fixed-effects logit regression results for unmet healthcare needs.

Variables	Odds ratio	95% CI
Marital status (married)	1.078	[0.813, 1.429]
Employment (manual)	0.941	[0.806, 1.099]
Employment (non-economic)	0.773^**^	[0.657, 0.909]
Region (metropolitan)	0.909	[0.559, 1.476]
Region (capital)	1.171	[0.731, 1.877]
Education (high school or below)	0.655	[0.316, 1.356]
Education (college or above)	0.687	[0.278, 1.695]
Health insurance (Medical aid)	0.827	[0.587, 1.165]
Health insurance (Other)	1.253	[0.902, 1.742]
Income (2nd Quintile)	0.792^***^	[0.697, 0.901]
Income (3rd Quintile)	0.724^***^	[0.621, 0.844]
Income (4th Quintile)	0.851	[0.719, 1.006]
Income (5th Quintile)	0.879	[0.730, 1.059]
No. of chronic diseases	1.003	[0.954, 1.054]
Disability status (yes)	0.852	[0.610, 1.190]
Activity limitation (yes)	1.677^***^	[1.481, 1.899]
Care recipient (yes)†	1.279^**^	[1.078, 1.517]
Korean medicine utilization (yes)†	1.022	[0.932, 1.121]
Care recipient × KM use†	0.895	[0.669, 1.199]
Year 2015 (ref. 2014)	1.086^*^	[1.004, 1.175]
Year 2016	0.801^***^	[0.737, 0.871]
Year 2017	0.765^***^	[0.703, 0.833]
Year 2018	0.916^*^	[0.842, 0.997]
Number of person-years observations	66,188 (23,956)	
Individuals contributing to estimation	5,165	
Year fixed effects	Yes	
Estimation method	Conditional maximum likelihood	

^*^ *p* < 0.05, ^**^ *p* < 0.01, ^***^ *p* < 0.001. † indicates key variables of interest. Reference categories: unmarried, non-manual employment, rural, ≤ middle school education, National Health Insurance, 1st income quintile, no disability, no activity limitation, no care need, no KM use, and year 2014. Estimates are odds ratios from a conditional fixed-effects logit model with year fixed effects; individual effects are conditioned out of the likelihood, which is identified by 5,165 individuals (23,956 person-years) exhibiting within-person variation in the outcome.

Among the covariates, activity limitation showed the strongest association with unmet healthcare needs (OR = 1.68, 95% CI: 1.48–1.90), indicating substantially higher odds of experiencing unmet needs among individuals with activity limitations. Non-economic employment status (OR = 0.77, 95% CI: 0.66–0.91) and the second- and third-income quintiles (OR = 0.79, 95% CI: 0.70–0.90; OR = 0.72, 95% CI: 0.62–0.84) were associated with lower odds of unmet healthcare needs. Other covariates — including region, education, health insurance type, number of chronic diseases, and disability — were not significantly associated with within-person changes in unmet healthcare needs. Unmet healthcare needs also varied significantly across survey years, being lower in 2016–2018 than in 2014.

Regarding the key variables of interest, receiving a care was significantly associated with higher odds of unmet healthcare needs (OR = 1.28, 95% CI: 1.08–1.52, *p* = 0.005), indicating that individuals receiving formal or informal care had approximately 28% higher odds of experiencing unmet healthcare needs than those not receiving care. KM utilization was not independently associated with unmet healthcare needs (OR = 1.02, 95% CI: 0.93–1.12). The interaction term between care receipt and KM utilization was directionally consistent with a buffering association — among care recipients, KM use corresponded to approximately 10% lower odds of unmet healthcare needs than expected from the independent associations alone — but did not reach statistical significance (OR = 0.90, 95% CI: 0.67–1.20). Point estimates for the key variables were virtually unchanged across the sensitivity analyses (Supplementary Table S1).

## 4 Discussion

This study investigated the moderating association of KM utilization in the relationship between an individual receiving formal or informal care and their unmet healthcare needs using five waves of nationally representative panel data. The findings reveal three key results: (1) being a care recipient is significantly associated with higher odds of unmet healthcare needs; (2) KM utilization is not independently associated with within-person changes in unmet healthcare needs; and (3) the interaction between receiving care and KM utilization is directionally consistent with a buffering association but does not reach statistical significance. These findings have important implications for healthcare policy and for the integration of traditional medicine into community-based care frameworks.

The central question of this study was whether KM utilization moderates the elevated risk of unmet healthcare needs among care recipients. The interaction between care receipt and KM use did not reach statistical significance (OR = 0.90, 95% CI: 0.67–1.20). Notably, however, the point estimate was directionally consistent with our hypothesis: among care recipients, those who used KM services in a given year had approximately 10% lower odds of reporting unmet healthcare needs than expected from the independent associations alone, whereas KM use was unrelated to unmet needs in the remainder of the sample. Given the width of the confidence interval, this pattern cannot be distinguished from chance and should be interpreted as suggestive rather than conclusive. Several mechanisms could nevertheless plausibly generate such a buffering association. Clinically, KM's holistic and patient-centered approach, which emphasizes comprehensive symptom management, alleviation of chronic pain, and maintenance of functional ability, aligns closely with the complex multi-morbidity profiles commonly observed among individuals receiving care (22).

KM is particularly well-suited to home-based and community-based care delivery, as its core modalities, including acupuncture, moxibustion, and manual therapy, rely primarily on practitioner skill rather than costly equipment, making them inherently adaptable to home-visit settings (22). For care recipients with severe mobility limitations, this capacity for in-home service delivery may be especially valuable, as it eliminates the need for travel to healthcare facilities. Consequently, for individuals whose ability to access healthcare is constrained by their reliance on care services, KM may reduce the risk of unmet healthcare needs.

Structurally, KM clinics in South Korea are widely distributed within local communities and may be more accessible for people with mobility limitations (23). Together with the recent policy initiatives such as community-based integrated care (Community Care) and pilot programs for home-visit KM services, these features may reduce both geographical and psycho-social barriers to accessing healthcare for individuals with severe mobility limitations (22). Nevertheless, as with other healthcare professions, regional disparities in workforce distribution may still affect access in some rural or underserved areas, highlighting the need for continued expansion of community-based and home-visit KM services to improve equitable access. Consequently, for care recipients whose ability to access healthcare is constrained, KM may bridge this gap by helping to meet their healthcare needs.

The protective association of KM utilization with unmet healthcare needs is also notable, even though not statistically significant, and may reflect the role of KM as a complementary pathway for healthcare access. Older adults commonly use KM for symptom relief, particularly for musculoskeletal conditions that can directly impact disability and activity limitation (16). KM services, including acupuncture, herbal medicine, and various manual therapies, may therefore help to address healthcare needs that are not fully met by conventional Western medicine (WM) alone (24). Although our analysis did not examine WM utilization, previous studies have reported that older adults and those with chronic diseases are more likely to use both KM and WM outpatient services than KM or WM alone. This finding is particularly relevant given that KM is widely accessible and funded by NHI, Medical Aid and private health insurance. KM may therefore serve as an additional source of care for individuals with chronic conditions and functional limitations, who constitute the majority of care recipients (25).

The finding that individuals receiving formal or informal care have significantly higher odds of unmet healthcare needs was not unexpected and is consistent with previous research from South Korea (7). This association may be explained by several mechanisms. Individuals who rely on formal or informal care services often experience substantial physical, mental health and/or cognitive decline and frequently live with multiple chronic conditions, which can create additional barriers to accessing conventional healthcare services (3). For these individuals, navigating a highly fragmented, specialized, and hospital-centered healthcare system is not only physically and emotionally demanding, but economically burdensome (11).

Also unsurprising was the finding that activity limitation was the strongest predictor of unmet healthcare needs (OR = 1.68). This is consistent with the broader literature on healthcare accessibility, which identifies functional limitations as a primary driver of unmet healthcare needs across populations (11, 18). The results affirm the importance of addressing mobility and functional barriers in healthcare delivery for care recipients. The association between the second- and third-income quintiles and lower unmet needs affirms the impact that financial factors have on healthcare access (26). Notably, several covariates that are strong correlates of unmet needs in cross-sectional studies, including region, education, health insurance type, chronic disease count, and disability, were not significantly associated with within-person changes in unmet needs in our fixed-effects specification. This is expected, because the fixed-effects design identifies associations only from within-person change over a five-year window and absorbs stable between-person differences; it should not be read as contradicting the cross-sectional literature. It also highlights the limitations of relying solely on self-reported unmet healthcare needs, which reflect individuals' perceived access to healthcare rather than objectively verified unmet medical needs. Such perceptions may be influenced by personal expectations, health literacy, and other psychosocial factors. Therefore, the findings should be interpreted as associations with perceived unmet healthcare needs rather than objectively measured healthcare access.

It is also important to interpret these findings within the context of South Korea's dual healthcare system. Although Korean Medicine and Western Medicine are both licensed professions covered under the National Health Insurance system, they are largely delivered through parallel practice structures with limited formal integration. Patients are able to utilize services from both systems, but standardized referral pathways, shared electronic medical records, and routine interdisciplinary communication between KM and WM practitioners remain limited. Consequently, vulnerable patients with complex health needs may experience fragmented care or uncoordinated treatment plans when receiving services from multiple providers. Therefore, the buffering association observed in this study should not be interpreted as evidence that the existing dual-practice system is fully integrated. Rather, our findings suggest that greater coordination between KM and WM, including shared care pathways, standardized referral mechanisms, and multidisciplinary community-based care models, may further reduce unmet healthcare needs among care recipients.

The integration of KM into mainstream healthcare also depends on the strength of its clinical evidence base. Although evidence supporting KM has grown substantially, variations in the quality of evidence across conditions continue to influence healthcare policy and institutional integration. Importantly, our findings should not be interpreted as evidence of clinical efficacy, but rather as indicating that KM utilization may serve as an additional pathway to reduce unmet healthcare needs among care recipients.

The findings from this study have several policy implications. First, they suggest that the integration of KM services into community-based care models is a policy option worth evaluating as a means of addressing unmet healthcare needs among individuals with care needs, although the present results do not yet provide confirmatory evidence of a buffering effect. Given the ongoing expansion of community care programs in South Korea, incorporating KM practitioners into multidisciplinary care teams could help address the healthcare needs of individuals with complex care needs and improve accessibility to services. Second, policies promoting care recipients' access to KM, such as expanding insurance coverage for KM services specific to care-related health conditions or establishing KM home-visit programs, may reduce financial barriers to accessing healthcare. Although many KM services are reimbursed under the National Health Insurance system, some treatments remain associated with out-of-pocket costs, which may limit accessibility for certain patients. Addressing these coverage gaps may further enhance equitable access to KM services among vulnerable populations. Third, the significant association of activity limitation affirms the importance of care delivery models that bring healthcare services to the patient rather than requiring patients to travel to healthcare facilities. In this regard, KM is well-suited to meet this need, as its core modalities, including acupuncture, moxibustion, and manual therapy, rely primarily on practitioner skill rather than costly equipment, making it inherently adaptable to primary care and home-visit settings. KM's tradition of home-based care may be particularly beneficial (2, 4).

This study addresses methodological limitations of previous cross-sectional studies examining the relationship between KM utilization and unmet healthcare needs that have been limited by their inability to control for unobserved individual heterogeneity, such as health-seeking behaviors, health literacy, and personal healthcare preferences, which in turn, may confound the unobserved associations (2, 4, 18, 19). By employing a FE panel regression model that examines within-individual changes over time, the approach controls for time-invariant individual characteristics, providing stronger longitudinal evidence than conventional cross-sectional analyses through control of time-invariant individual characteristics. Therefore, compared with prior cross-sectional research, the findings of this study provide stronger longitudinal evidence regarding the association between KM utilization and unmet healthcare needs.

Notwithstanding these strengths, this study has several limitations that should be acknowledged. First, while the fixed-effects model controls for time-invariant individual characteristics, it cannot account for time-varying confounders that may simultaneously influence both KM utilization and unmet healthcare needs. Changes in health status, symptom severity, care intensity, family support, or healthcare access barriers may influence both KM utilization and unmet healthcare needs. Therefore, the observed associations should not be interpreted as causal effects. Second, the binary measure of KM utilization does not capture the intensity, type, or frequency of KM services used, which may have heterogeneous associations with unmet healthcare needs; such aggregation is also likely to bias interaction estimates toward the null. Third, the statistical power to detect a moderating association was limited: care recipients constituted only about 3% of the sample, and fixed-effects identification further restricts estimation to individuals whose unmet-need status changed over time. The confidence interval for the interaction (0.67–1.20) indicates that only relatively large buffering associations could have been detected reliably; the null interaction should therefore be read as an absence of evidence rather than evidence of absence, and interaction terms in nonlinear models must additionally be interpreted on the multiplicative odds scale. Fourth, the operational definition of care recipient combined informal and formal care, which may carry different meanings, formal care may indicate more severe functional limitations and institutional selection, whereas informal care may reflect family support and social capital, and could therefore obscure mechanisms operating in opposite directions. The limited number of care recipients with within-person variation in the outcome precluded reliable disaggregation by care type, intensity, or setting within the fixed-effects framework; this definition also assumes that those not receiving care did not need care. Fifth, because identification relies on individuals whose unmet-need status changed over time, the estimates pertain to this subpopulation and may not generalize to adults whose unmet healthcare needs remain stable over time. Sixth, unmet healthcare needs were measured based on self-reported responses and may therefore be subject to recall bias or differential reporting across population subgroups. Seventh, this study did not include WM utilization as a covariate or examine the interaction between KM and WM use. As a result, it is not possible to determine whether the association of KM utilization operates independently of WM use, or whether KM users who concurrently utilize WM services experience differential associations with unmet healthcare needs compared to those using KM or WM exclusively. Future research should adopt a polytomous classification of healthcare utilization (e.g., KM only, WM only, both KM and WM, neither) to disentangle these potentially heterogeneous associations. Finally, the study period (2014–2018) precedes significant policy changes in community care and visiting medical services that were implemented after 2019. Consequently, the findings may not fully reflect the current policy environment. Future research should investigate whether similar moderating associations are observed with more detailed measures of KM utilization, larger samples of care recipients (for example, National Health Insurance claims data linked to survey information), and in the context of recent care policy reforms.

## 5 Conclusion

This study provides longitudinal evidence that adults who receive formal or informal care are more likely to report unmet healthcare needs, even within a system with universal health coverage. The association between KM utilization and lower unmet healthcare needs among care recipients was directionally consistent with a buffering role but did not reach statistical significance, and should therefore be regarded as a hypothesis warranting confirmation rather than an established effect. In the context of rapid population aging and growing care demands, these findings highlight the persistent access barriers faced by care recipients and suggest that KM could function as a complementary component of community-based care. As South Korea continues to expand its community-based integrated care infrastructure, future research should further investigate the mechanisms through which KM utilization may reduce unmet healthcare needs, including dose–response measures of KM use and the interplay between KM and WM utilization patterns among care recipients, and evaluate the cost-effectiveness of incorporating KM into comprehensive care programs.

## Data Availability

The data used in this study are available from the Korea Health Panel Survey (KHPS) upon reasonable request and permission from the data provider.
