# Supplementary material for: Buffering the healthcare gap: the role of Korean medicine in unmet healthcare needs among care recipients
Source: Front Med (Lausanne). 2026 Aug 25;13:1890354. doi: 10.3389/fmed.2026.1890354 (PMC13549865; doi:10.3389/fmed.2026.1890354)
Supplement: Supplementary file 1 [file Table_1.docx]

**Supplementary Table S1. Robustness of the key estimates across estimators**

Odds ratios (95% CI). All models include the full covariate set of Table 2.

|  | **(A) Conditional FE logit (main model)** | **(B) Unconditional FE logit, unweighted** | **(C) Unconditional FE logit, weighted** |
| --- | --- | --- | --- |
| Care recipient | 1.279 (1.078–1.517)** | 1.315 (1.056–1.638)* | 1.282 (0.986–1.666) |
| KM utilization | 1.022 (0.932–1.121) | 1.026 (0.912–1.154) | 0.966 (0.838–1.114) |
| Care × KM | 0.895 (0.669–1.199) | 0.888 (0.610–1.291) | 0.901 (0.576–1.409) |
| **Individual FE** | Conditioned out | Yes (dummies) | Yes (dummies) |
| **Year fixed effects** | Yes | No | Yes |
| **Survey weights** | No | No | Normalized (mean = 1) |
| **Standard errors** | Conditional MLE | Model-based | Clustered (individual) |
| **Person-years (input)** | 66,188 | 23,956 | 22,842 |
| **Individuals identifying estimates** | 5,165 | 5,165 | 4,885 |

Notes: (A) Conditional (Chamberlain) fixed-effects logit with year dummies, unweighted — main specification reported in Table 2. (B) Unconditional fixed-effects logit, unweighted, without year effects. (C) Unconditional fixed-effects logit with individual and year fixed effects, KHPS longitudinal weights normalized to a mean of one, and standard errors clustered at the individual level; 3,655 person-years with zero longitudinal weight are excluded. * p < 0.05, ** p < 0.01, *** p < 0.001.
